# Space-by-time manifold representation of dynamic facial expressions for emotion categorization

**DOI:** 10.1167/16.8.14

**Published:** 2016-06-15

**Authors:** Ioannis Delis, Chaona Chen, Rachael E. Jack, Oliver G. B. Garrod, Stefano Panzeri, Philippe G. Schyns

**Affiliations:** ioannis.delis@columbia.edu https://sites.google.com/site/ioannisdeliswebpage/; c.chen.1@research.gla.ac.uk http://www.psy.gla.ac.uk/staff/?id=CC002; rachael.jack@glasgow.ac.uk http://www.gla.ac.uk/schools/psychology/staff/rachaeljack/; oliver.garrod@glasgow.ac.uk http://www.gla.ac.uk/researchinstitutes/neurosciencepsychology/staff/olivergarrod/; stefano.panzeri@iit.it http://cncs.iit.it/people/stefano-panzeri.html; philippe.schyns@glasgow.ac.uk http://www.gla.ac.uk/researchinstitutes/neurosciencepsychology/staff/philippeschyns/; Institute of Neuroscience and Psychology, School of Psychology, University of Glasgow, Glasgow, UK; Department of Biomedical Engineering, Columbia University, New York, NY, USA; School of Psychology, University of Glasgow, Glasgow, UK; Laboratory of Neural Computation Center for Neuroscience and Cognitive Systems @UniTn, Istituto Italiano di Tecnologia, Rovereto, Italy

**Keywords:** *emotion categorization*, *space-by-time manifold*, *nonnegative matrix factorization*, *NMF*, *mental representations*, *dynamic facial expressions*

## Abstract

Visual categorization is the brain computation that reduces high-dimensional information in the visual environment into a smaller set of meaningful categories. An important problem in visual neuroscience is to identify the visual information that the brain must represent and then use to categorize visual inputs. Here we introduce a new mathematical formalism—termed space-by-time manifold decomposition—that describes this information as a low-dimensional manifold separable in space and time. We use this decomposition to characterize the representations used by observers to categorize the six classic facial expressions of emotion (happy, surprise, fear, disgust, anger, and sad). By means of a Generative Face Grammar, we presented random dynamic facial movements on each experimental trial and used subjective human perception to identify the facial movements that correlate with each emotion category. When the random movements projected onto the categorization manifold region corresponding to one of the emotion categories, observers categorized the stimulus accordingly; otherwise they selected “other.” Using this information, we determined both the Action Unit and temporal components whose linear combinations lead to reliable categorization of each emotion. In a validation experiment, we confirmed the psychological validity of the resulting space-by-time manifold representation. Finally, we demonstrated the importance of temporal sequencing for accurate emotion categorization and identified the temporal dynamics of Action Unit components that cause typical confusions between specific emotions (e.g., fear and surprise) as well as those resolving these confusions.

## Introduction

All categorization problems start with a high-dimensional external world that must be reduced to a small number of equivalence classes called categories. Categorization of complex visual stimuli typically comprises two stages. A first stage projects the high-dimensional input onto a low-dimensional space that captures the main covariations of the input. The second stage uses this projection, together with a classifier, to categorize the input. However, in most complex categorization problems, the low-dimensional representation is unknown, and so not only must it be discovered, it must be valid when it is used to model human categorization (e.g., in computer vision or social robotics applications). To resolve this problem, we introduce a computational framework and apply it to discover how the brain resolves a biologically important categorization: the categorization of the six classic facial expressions of emotion from dynamic facial expressions (Jack & Schyns, 2015).

Our approach first characterizes the individual observer's mental representations of each facial expression as a combination of a small set of spatial and temporal dimensions that we call the categorization manifold (Seung & Lee, 2000). To learn this representation, we build on previous research on muscle synergies (d'Avella, Saltiel, & Bizzi, 2003; Delis, Panzeri, Pozzo, & Berret, 2014; Tresch, Cheung, & d'Avella, 2006) to introduce a novel method, which we call the space-by-time manifold, and identify a coordinate set that describes the categorization manifold. In brief, this method is based on the principles of nonnegative matrix factorization (NMF; Lee & Seung, 1999), it makes the assumption that the categorization manifold is separable in space and time (Delis et al., 2014), and it uses linear discriminant analysis (LDA; Delis, Berret, Pozzo, & Panzeri, 2013b; Duda, Hart, & Stork, 2001) to identify the dimensions of the perceptual space that are most useful for visual categorization. With this space-by-time manifold decomposition, we address important questions in emotion communication using facial expressions. In particular, we determine which synergistic facial movements communicate emotions, and we isolate the specific movements that cause confusions between specific emotion categories (e.g., fear and surprise; see Ekman, 1992; Gagnon, Gosselin, Hudon-ven Der Buhs, Larocque, & Milliard, 2010; Jack, Blais, Scheepers, Schyns, & Caldara, 2009; Matsumoto & Ekman, 1989; Moriguchi et al., 2005; Roy-Charland, Perron, Beaudry, & Eady, 2014) from those that resolve these confusions. That is, we can identify the low-dimensional space-by-time representation that predicts categorization behavior.

Having characterized the categorization manifold, we then validate its plausibility and test the importance of its dynamic temporal structure. We show that temporal dynamics significantly affect the accuracy and speed of emotion categorization. We also uncover the temporal sequences of facial expressions that optimize emotion categorization, thus serving as optimal signals to communicate the six classic emotions.

Such identification of basic components for decoding as well as generating optimal social signals can be a useful computational tool in computing and graphical environments. For example, in machine vision the synergies of facial movements combined with their temporal dynamics can be used as a set of priors to facilitate identification of emotions from dynamic facial expressions. Another field in which our method can find potential applications is social robotics and digital avatars, where current research aims to generate face signals that are psychologically valid, perceptually plausible, and minimally confusable. The manifold dimensions extracted here can be used as the generative basis of such social face signals because (a) they derive from the mental representations of individual observers and are thus de facto perceptually valid and (b) we further demonstrate how to select the most diagnostic manifold regions to design minimally confusable signals.

## Experiment 1: Emotion categorization of random facial movements

To gather empirical data needed for the characterization of the space-by-time categorization manifold, we first conducted an extensive subjective perception task in which observers categorized random facial movements according to the six classic emotions.

### Observers

We recruited 60 White Western observers (59 European, one North American; 31 women, 29 men; mean age = 22 years, *SD* = 1.71 years) with normal or corrected-to-normal vision and minimal exposure to and engagement with non-Western cultures (De Leersnyder, Mesquita, & Kim, 2011) as assessed by questionnaire (see Supplementary Material: Observer Questionnaire). All observers gave written informed consent and received 6 pounds per hour. The Glasgow University College of Science and Engineering Ethics Committee provided ethical approval.

### Stimuli

**Figure 1:**
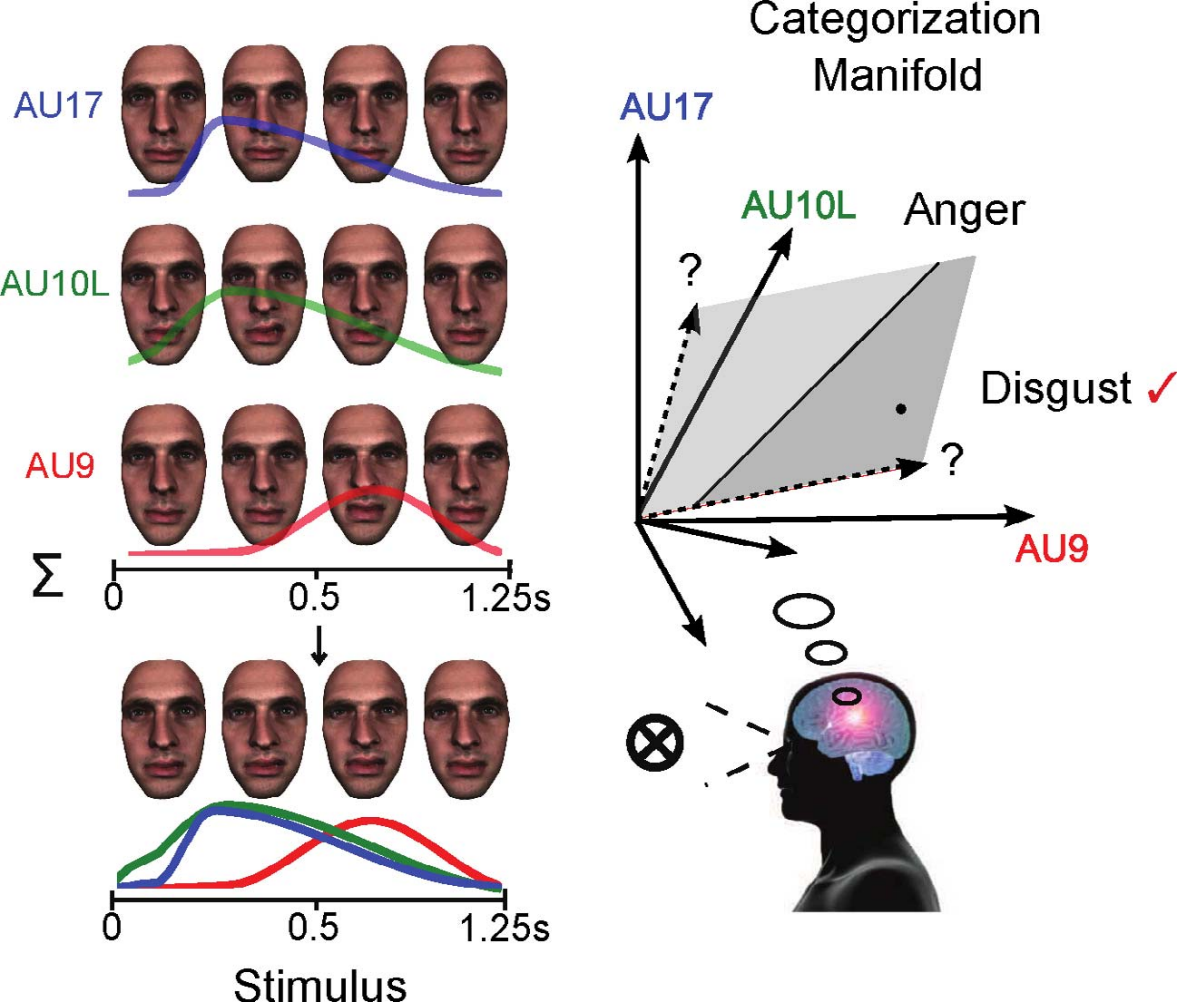
Identifying the low-dimensional categorization manifold of dynamic facial expressions of emotion. Stimulus: On each experimental trial, the GFG randomly selected a subset of AUs—here Upper Lid Raiser (AU5) color-coded in red, Nose Wrinkler (AU9) in green, and Upper Lip Raiser (AU10) in blue—and assigned a random movement to each AU using six parameters (onset latency, acceleration, peak amplitude, peak latency, deceleration, and offset latency—see labels illustrating the red temporal-activation curve). The dynamic AUs are then combined to produce a photorealistic random facial animation, illustrated here with four snapshots across time. Categorization manifold: As illustrated with our geometric interpretation, observers categorized the random facial animation as one of the six classic emotions—happy, surprise, fear, disgust, anger, and sad—if the facial movements projected onto the corresponding categorization-manifold region (e.g., the black dot in the dark-gray anger region) or selected “other.” Our aims are thus twofold: (a) to identify the axes of the lower dimensional categorization manifold (indicated with dashed lines and “?”) and (b) to characterize the decision boundary for categorization given the new geometry (indicated here with the black line separating the light- and dark-gray manifold regions of disgust and anger, respectively).

Figure 1 illustrates the stimulus generation and task procedure. On each experimental trial, a Generative Face Grammar (GFG; Yu, Garrod, & Schyns, 2012) randomly selected from a set of 42 core Action Units (AUs)—specific face movements described by the Facial Action Coding System (Ekman, Friesen, & Hagar, 1978; for a full list and more details, see Supplementary Material)—a subsample of AUs from a binomial distribution (*n* = 5, *P* = 0.6, minimum = 1, maximum = 6, median = 3). In this illustrative example trial, the GFG selected Upper Lid Raiser (AU5) color-coded in red, Nose Wrinkler (AU9) color-coded in green, and Upper Lip Raiser (AU10) color-coded in blue. For each AU separately, the GFG selected random values for each of six temporal parameters (onset latency, acceleration, peak amplitude, peak latency, deceleration, and offset latency—see labels illustrating the red temporal-activation curve) from a uniform distribution. The GFG then combined the random dynamic AUs to create a random photorealistic facial animation displayed on a unique same-race face identity generated using standard procedures (Yu et al., 2012). We rendered all facial animations using 3ds Max. We refer the reader to the Supplementary Material: Stimulus Parameter Sampling for more details on the parameter-sampling procedures.

### Procedure

On each experimental trial, observers categorized the random facial animation according to the six classic emotions—happy, surprise, fear, disgust, anger, and sad—when the random facial movements projected onto the categorization manifold region corresponding to one of the emotion categories (e.g., Jack, Garrod, Yu, Caldara, & Schyns, 2012). In Figure 1, we depict the representation of each emotion as a low-dimensional subspace (or manifold) lying in the high-dimensional space (42 × 6 dimensions, shown in Figure 1 as axes with solid lines) of dynamic AU activations. Alternatively, if none of the available response options accurately described the facial animation, observers selected “other.” In this illustrative example, the dashed axes represent a manifold region that discriminates disgust (light gray) from anger (dark gray). The black dot represents the facial-expression stimulus on this trial, which falls into the dark-gray region—hence the observer selected anger, as indicated by the red tick mark. We presented stimuli on a black background displayed on a 19-in. flat-panel Dell monitor with a refresh rate of 60 Hz and resolution of 1024 × 1280 pixels. All stimuli appeared in the center of the observer's visual field, played only once (duration of 1.25 s), and remained visible until response. A chin rest ensured a constant viewing distance of 68 cm, with images subtending 14.25° (vertical) by 10.08° (horizontal) of visual angle, reflecting the average size of a human face (Ibrahimagić-Šeper, Čelebić, Petričević, & Selimović, 2006) during natural social interaction (Hall, 1966). We randomized the order of the facial animations and face gender across each observer and controlled stimulus presentation and observer responses using MATLAB 2009b.

For each observer, this procedure produced single-trial pairings between a facial-expression animation (i.e., dynamic AUs) and the observer's emotion-categorization response. We now present a methodology that uses these single-trial pairings to derive the low-dimensional subspaces that categorize and discriminate each emotion (in Figure 1, see the black line that discriminates disgust [light gray] from anger [dark gray]).

### Analyses

#### Space-by-time NMF decomposition of dynamic facial-expression representations

To identify the dynamic facial-expression patterns that reliably and distinctly describe the six classic emotions, we introduce the *space-by-time manifold*—a dimensionality-reduction algorithm based on NMF (Lee & Seung, 1999). The space-by-time manifold represents all facial movements (described on each trial by an *S* AUs × *T* temporal parameters matrix; here *T* = 6, *S* = 42) using a set of nonnegative spatial (AU) components and a set of nonnegative temporal components (describing the temporal profile of each AU activation). To approximate each single-trial facial-expression stimulus, the AU and temporal components are linearly combined using scalar activation coefficients. Formally, the AU activity **M***_n_* with dimensions (*T* × *S*) recorded during one trial *n* is factorized as follows (Delis et al., 2014, 2015):

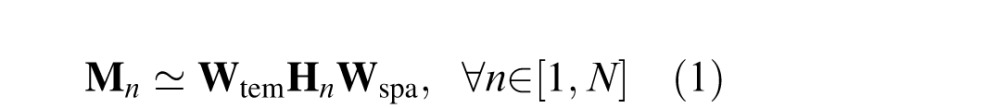
where **W**_tem_ is a (*T* × *P*) matrix whose columns are the temporal components, **W**_spa_ is an (*L* × *S*) matrix whose rows are the AU components, and **H***_n_* is a (*P* × *L*) matrix containing the coefficients that combine each of the *P* temporal components with each of the *L* spatial ones.

Hence, for each observer we formed a single-trial dynamic AU matrix **M**(*T* × *S* × *N*), where *N* is the number of trials (here *N* = 2,400). We used this matrix as input to the space-by-time decomposition algorithm (Delis et al., 2014)—a MATLAB implementation is available online at https://sites.google.com/site/ioannisdeliswebpage/software/sNM3F.zip—to extract the AU and temporal components of facial movement that subsume each observer's emotion categorizations. Each AU component represents a specific conjunction of AUs, each temporal component represents a temporal profile of activations, and the linear combinations of AU and temporal components recode each emotion category in the manifold.

To summarize results, we then pooled the data matrices from all 60 observers (*N* = 2,400 × 60 in this case) and applied the same decomposition. We quantified similarity between each single-observer decomposition and the decomposition of the pooled data using the correlation coefficients between pairs of components. We found high similarity between the components of the single-observer decomposition and those of the pooled single-observer data (average correlation across components and observers was 0.97 ± 0.01 for the temporal components and 0.83 ± 0.03 for the AU components), thereby lending support to their consistency.

The space-by-time manifold decomposition thus reduces the high-dimensional categorization problem (here, 42 AUs × 6 temporal parameters = 252 degrees of freedom) to a low-dimensional representation. To select the number of informative dimensions, we performed a categorization analysis. Specifically, we used the single-trial coefficients **H***_n_* of the space-by-time manifold decomposition as inputs to an LDA to predict the emotion the observers categorized on each trial using a leave-one-out cross-validation procedure (Duda et al., 2001; Quian Quiroga & Panzeri, 2009). After evaluating categorization accuracy with *P* = 1 temporal and *N* = 1 spatial component, we iteratively added components and compute the categorization power of the resulting decompositions for the six emotion categories. Adding temporal and AU components translates into more temporal bursts and groups of AUs carrying emotion-categorization information respectively. We stopped adding components when significant increases in categorization performance (percent correct classification, *p* < 0.001) stopped. From this procedure, the chosen set of *N* spatial and *P* temporal components is the smallest decomposition that carries the highest categorization power (>70% on average) of the six emotion categories (Delis et al., 2013a, 2013b).

We chose to decompose this data set with NMF for several reasons. The main reason is the constraint of nonnegativity imposed by NMF on both the extracted components and the coefficients. This property is useful to ensure that all components are directly interpretable as dynamical facial expressions, because face movements cannot be activated “negatively.” The nonnegative coefficients allow only additive, not subtractive, combinations of components—a decomposition compatible with the intuitive notion that components act as parts in a categorization task. Moreover, nonnegativity naturally leads to low-dimensional sparse representations with typically a small number of components (because they do not allow positive and negative terms canceling each other out; Lee & Seung, 1999). The second reason was that, unlike other methods such as principal-component analysis (PCA) or independent-component analysis (ICA), NMF does not impose additional constraints (such as, e.g., orthogonality or independence) on statistical relationship between the components; this was particularly useful for this application in which these assumptions would not seem compatible with the partly overlapping nonnegative facial-expression patterns that communicate emotions. The third reason was the simplicity of the resultant NMF decomposition and the fact that the original facial-movement data can be straightforwardly reconstructed by a linear combination of the extracted components. This makes it—in principle—possible to generate simply and linearly new facial expressions based on an NMF decomposition, thereby complementing other proposals for facial-expression generation based on nonlinear dimensionality-reduction methods, such as manifold learning (Martinez & Du, 2012).

**Figure 2:**
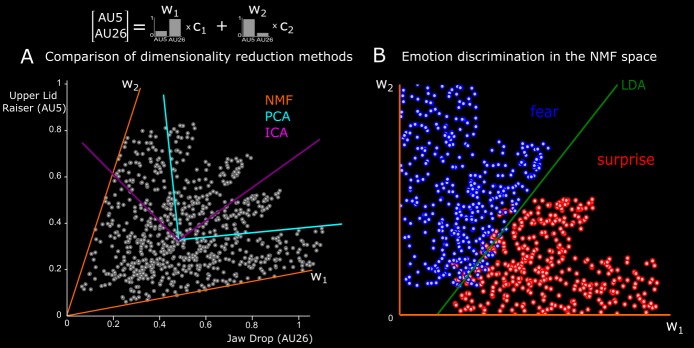
Illustrative application of our method to simulated AU data. (A) Comparison of dimensionality-reduction methods. Here we show how three dimensionality reduction methods (NMF, color-coded in orange; PCA, color-coded in cyan; and ICA, color-coded in magenta) recode a simulated data set comprising the activations of two AUs—Upper Lid Raiser (AU5) on the y-axis and Jaw Drop (AU26) on the x-axis. Data are generated by the linear combination of two basis functions w_1_ and w_2_ that represent two functional dimensions. The first dimension consists of a low activation of Upper Lid Raiser and a high activation of Jaw Drop; the second, vice versa (see bars). Gray spheres reflect individual trials (600 in total) represented with different coefficients (c_1_ and c_2_) for the basis functions. On half of the trials, we impose correlations (*p* = 0.7) between c_1_ and c_2_ to represent AU synergies. NMF recovers the correct basis functions (see orange axes). In contrast, neither PCA (cyan axes) nor ICA (magenta axes) recovers the original basis, because of their underlying assumptions. PCA starts from the dimension explaining the most variance, adding one orthogonal dimension, and ICA looks for independent dimensions. For both PCA and ICA, the second dimension comprises negative values for one of the two AUs, which is incompatible with their functional role as representations of AU activations. (B) Emotion discrimination in the NMF space. In the space defined by w_1_ and w_2,_ we apply LDA to discriminate trials categorized as fear (blue spheres) from those categorized as surprise (red spheres). LDA determines the categorization boundary (green line) that reliably discriminates the two emotions (93% correct discrimination).

We show in Figure 2A an illustrative comparison of PCA, ICA, and NMF when applied to simulated AU data. In this example, data are generated by the linear combination of two AU components (w_1_ and w_2_), where w_1_ consists of a low activation of Upper Lid Raiser (AU5) and a high activation of Jaw Drop (AU26), and vice-versa for w_2_ (see bars). Gray spheres reflect individual trials (600 in total) represented by two coefficients (c_1_ and c_2_) that linearly combine the two components. On half of the trials, we impose correlations (*p* = 0.7) between c_1_ and c_2_ to represent AU synergies. NMF is shown to correctly recover the simulated dimensions, whereas PCA starts from the dimension explaining the most variance, adding one orthogonal dimension, and ICA looks for statistically independent dimensions. As a result, PCA and ICA identify components with negative values for one of the two simulated AUs, which is inconsistent with their functional role as representations of AU activations.

#### Decoding analysis to identify discriminating dimensions

The foregoing analysis delivered a few AU and temporal components that describe the six classic emotion categories. Here, we determined which of these components contribute to discriminate the pairs of emotions that are often confused (e.g., fear and surprise; Ekman, 1992; Gagnon et al., 2010; Roy-Charland et al., 2014). To this aim, we performed an emotion-decoding analysis using the *P* × *L* coefficients of the space-by-time decomposition.

To identify the combinations of AU components and temporal components that discriminate a given emotion from all others, we performed LDA (in a leave-one-out cross-validation scheme) on the *P* × *L*–dimensional space (Duda et al., 2001). We input each activation coefficient of the space-by-time decomposition to LDA and computed the discrimination power (percent correct decoding) carried by each combination of AU and temporal components.

Then we computed the discrimination power of the AU dimension and the temporal dimension separately. We present this procedure in detail for the AU components; the same analysis applies to the temporal components. We described each AU component with the activation coefficients that combine it with the *P* temporal components, resulting in *P* × *L* parameters on an *L*-dimensional space. LDA determined the linear boundaries that split the *L*-dimensional space into subspaces corresponding to each emotion. We found a discrimination performance higher than 75% for the resulting subspaces (as computed by LDA) for all pairs of emotions. We show in Figure 2B an illustrative example of the application of LDA to simulated fear and surprise trials on the space defined by the NMF components. Here LDA is shown to determine a categorization boundary that discriminates fear from surprise.

### Results

#### AU and temporal dimensions for emotion categorization

**Figure 3:**
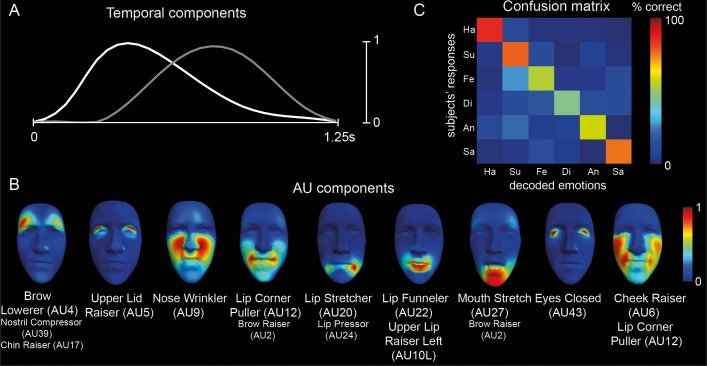
The space-by-time manifold decomposition for the categorization of the six classic emotions. (A) Temporal components. Temporal waveforms show the two distinct temporal components, with a total duration of 1.25 s. (B) AU components. Color-coded faces represent the nine AU components, with red indicating higher activations and blue indicating lower activations (see color bar to the right). Correspondingly, larger AU labels below each face represent the AUs with higher activation. As shown by the color-coding, the nine AU components represent relative levels of simultaneous activations of different AUs. (C) Confusion matrix. The color-coded matrix shows the accuracies (see squares across the diagonal) and confusions (averaged across observers) for the discrimination of the six emotions along the space-by-time manifold.

We found that nine AU components and two temporal components captured the information carried by the facial animations that was necessary to discriminate the six emotions. In other words, this basis was the smallest set of patterns of AU activations and temporal profiles that allowed the most accurate decoding of the emotion categories as judged by the observers. In Figure 3A, time-varying curves represent the temporal components, showing two distinct bursts of AU activations with different temporal profiles. The early temporal component (white line) begins at 0 ms, has a fast acceleration, peaks at approximately 415 ms, and decelerates slowly, whereas the late component (gray line) begins at approximately 375 ms and peaks at 750 ms, thereby following a more symmetric temporal profile in terms of acceleration and deceleration. The two temporal components are reminiscent of the two phases of hierarchically transmitted facial-expression information ( “biologically basic-to-socially specific”) reported previously (Jack, Garrod, & Schyns, 2014). In Figure 3B, color-coded faces represent the nine AU components (e.g., Upper Lid Raiser [AU5]; see AU labels below each face), where larger AU labels represent AUs with higher activations. The nine AU components represent relative levels of simultaneous activations of different AUs—i.e., synergies between facial movements. We decoded each observer's emotion categorization on each trial from the coefficients of the corresponding facial expression along this manifold. In Figure 3C, the color-coded matrix shows the categorization accuracies and confusions for the six-emotion discrimination, averaged over all observers and trials, indicating an average accuracy of 66% ± 1% across the six emotions.

To demonstrate the usefulness of reducing the data dimensionality by identifying the space-by-time manifold and then using its coordinates to express the facial movements, we compared this result with the emotion-categorization accuracy that we would obtain working directly on the raw time series of activation of all AUs. To compute this, we input the single-trial stimulus matrix of an example observer to LDA to decode the six emotions. We obtained 25% correct categorization, which was significantly lower than the categorization accuracy of the space-by-time manifold decomposition for this observer (65%, *p* < 0.001). This result indicates that the identified manifold effectively captures the informative dimensions of the facial-expression signals, and suggests that identifying a compact yet highly informative representation is crucial for the reliable categorization of the data. We then analyzed how these different patterns of activation contribute to the discrimination of specific pairs of emotions.

#### Surprise- versus fear-discriminating subspaces

**Figure 4:**
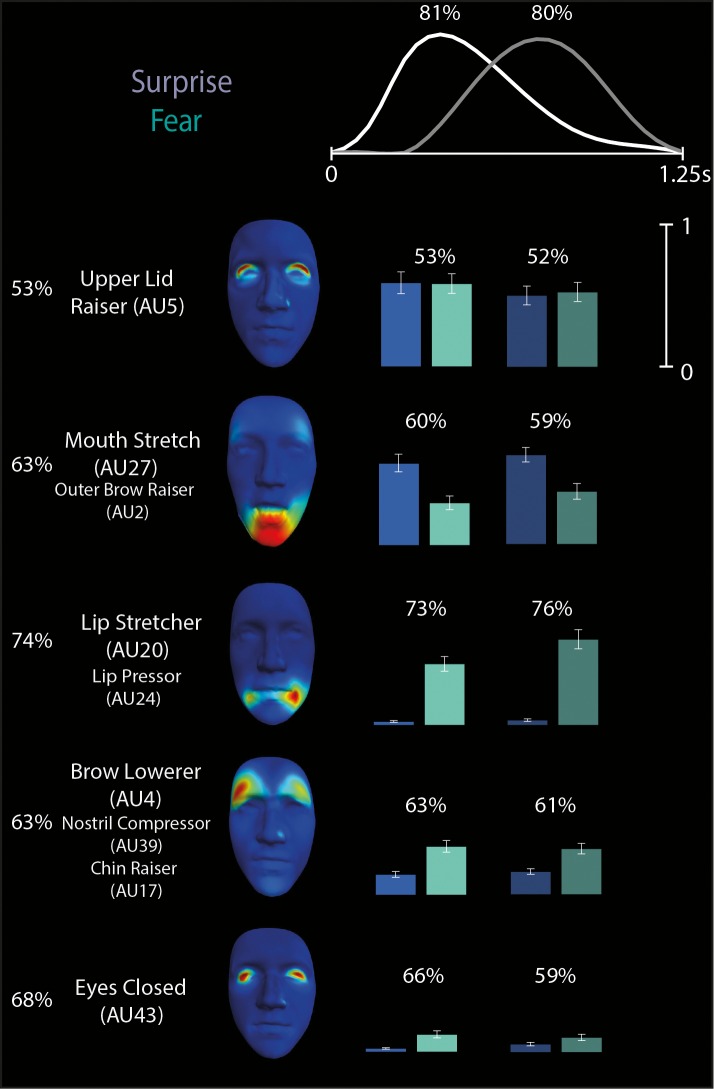
Space-by-time decomposition for surprise/fear distinction. The space-by-time decomposition identified two two temporal components (see waveforms in top) and five AU components (see color-coded faces and AU labels). Color-coded bars (blue = surprise, cyan = fear, with bright colors representing activations of the first temporal component and darker colors representing activations of the second temporal component) show the activation coefficients (trial averages ± standard error of the mean) that linearly combine each temporal component with each AU component. Reported percentages correspond to the average decoding accuracy (percent correct) across observers of the spatial components (left), temporal components (top), and space-by-time linear combinations of components (on top of each color-coded pair of bars). Chance-level decoding is 50%.

We first considered the components that are relevant to discriminating surprise from fear, a well-known confusion (Ekman, 1992; Gagnon et al., 2010; Roy-Charland et al., 2014). In Figure 4, we show the five AU components (left) and two temporal components (top) that combine to communicate surprise or fear (we excluded components with average activation coefficients lower than 10% of the maximal activation for these emotions). Color-coded bars organized in five rows (corresponding to the five AU components) and two columns (corresponding to the two temporal components) show the average activation coefficients combining the temporal and AU components for the confused emotion categories (blue: surprise, cyan: fear), where brighter colors correspond to activations of the first temporal component and darker colors correspond to activations of the second temporal component. Above each set of bars, percentages represent the decoding power of each combination of spatial and temporal components (chance level is 50% here and in all subsequent pair-wise discriminations). For example, the Lip Stretcher–Lip Pressor (AU20-24) coefficients indicate that the activated AU component corresponds mainly with trials categorized as fear (cyan bars supersede blue bars) and is on average more active later in the time course than early on (the second cyan bar, corresponding to the second temporal component, is higher than the first). Notably, our method groups AUs from different face regions (e.g., Mouth Stretch–Outer Brow Raiser, AUX-Y) into a single component, suggesting that the distinction between different emotions such as surprise and fear is achieved by considering a small number of relatively stereotyped and “synergistic” patterns of AU activation that resemble the muscle synergies often described in the theory of neural motor control (Alessandro, Delis, Nori, Panzeri, & Berret, 2013; Bizzi & Cheung, 2013; Bizzi, Cheung, d'Avella, Saltiel, & Tresch, 2008; Tresch, Saltiel, & Bizzi, 1999).

We then examined the surprise-versus-fear discrimination power of each component separately. For each AU component, we decoded the emotion using the coefficients of both the early and late temporal components. As suggested by the decoding accuracy of the AU components (see percentages to the left of each component in Figure 4), Lip Stretcher–Lip Pressor (AU20-24) contributes most to surprise-versus-fear discrimination (74% ± 2%), whereas Upper Lid Raiser (AU5) does not discriminate the two emotions (53% ± 1%), as its coefficients are comparable across both. Similarly, for each temporal component, we decoded the emotion using the coefficients of all five AU components. We found (see percentages on top of each temporal component in Figure 4) that the two temporal components had highly similar discrimination power (81% ± 2% vs. 80% ± 2%).

To identify the AU and temporal dimensions that are responsible for discriminating each emotion from all the others, we performed a decoding analysis on the 9 × 2–dimensional space. We used the activation coefficients of the space-by-time manifold decomposition to determine which AU components and temporal components contribute to the reliable categorization of each emotion. We applied LDA on the full 9 × 2–dimensional space of AU components to find the emotion-discrimination boundaries.

**Figure 5:**
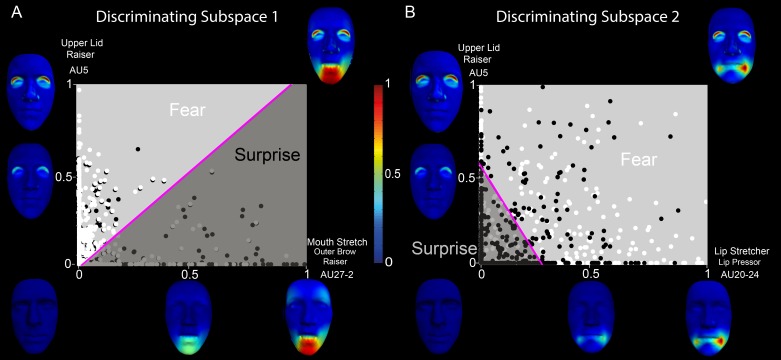
Discriminating emotion-category subspaces on the low-dimensional AU subspace—two examples of discriminating combinations of AU components. (A) Discriminating Subspace 1: Categorization of fear versus surprise on two AU dimensions—i.e., Upper Lid Raiser (AU5) and Mouth Stretch–Outer Brow Raiser (AU27-2). Color-coded spheres represent single-trial weightings of the two components (white = fear, black = surprise). The linear boundary (magenta line) obtained by an LDA splits the space into a fear (light gray) and a surprise (dark gray) region. Along each axis, color-coded faces show the corresponding face movements (red indicates the highest magnitude of vertex movement—see color bar in center) that represent the manifold dimensions at the origin (0, neutral face), midpoint (0.5, half of the maximal amplitude), and endpoint (1, maximal amplitude). The top right corner face illustrates the two AU components combined—i.e., Upper Lid Raiser (AU5) and Mouth Stretch–Outer Brow Raiser (AU27-2). B. Discriminating Subspace 2: Categorization of fear and surprise on two other AU components—i.e., Upper Lid Raiser (AU5) and Lip Stretcher–Cheek Raiser Left (AU20-6L).

We first examined the outcome of the LDA for discrimination of fear versus surprise. In Figure 5A the x- and y-axes represent two example AU components informative of fear (light gray) versus surprise (dark gray) discrimination (i.e., respectively, Upper Lid Raiser, AU5, and Mouth Stretch–Outer Brow Raiser, AU27-2). Color-coded faces along each axis show the corresponding face movements. Color-coded spheres show each observer's fear (white) and surprise (black) single-trial categorization responses, where the coordinates of each dot represent the respective contribution (i.e., linear weights) of both AU components to each categorization response. A linear categorization boundary (represented by the magenta line) splits the 2-D space, revealing two diagnostic regions—one corresponding to fear (light gray), comprising the Upper Lid Raiser (AU5), and the other corresponding to surprise (dark gray), comprising the Mouth Stretch–Outer Brow Raiser (AU27-2). This partition predicts that Mouth Stretch–Outer Brow Raiser (AU27-2) reliably categorizes surprise, whereas Upper Lid Raiser (AU5) categorizes fear. It also predicts that their combination should elicit confusions between the two emotion categories.

In Figure 5B, we present another 2-D subspace defined by Upper Lid Raiser (AU5) and Lip Stretcher–Cheek Raiser Left (AU20-6L), respectively. Here, LDA predicts that low weights on each component categorize the input facial expression as surprise, whereas a high weighting of both categorizes it as fear.

In sum, Upper Lid Raiser (AU5) is common across surprise and fear, thus leading to confusions. Combining Upper Lid Raiser (AU5) with the Lip Stretcher–Cheek Raiser Left (AU20-6L) component discriminates fear from surprise, whereas the Mouth Stretch–Outer Brow Raiser (AU27-2) component discriminates surprise from fear. The identified AUs are consistent with emotion recognition from facial expressions as defined using the Facial Action Coding System (Ekman, 1992), which validates the effectiveness of our approach in specifying the AUs that are shared across emotions as well as those that are emotion specific. The main difference from standard results is the activation of Mouth Stretch (AU27) instead of Jaw Drop (AU26), which can be explained by the high physical similarity between these two AUs (i.e., Mouth Stretch is a more intense form of Jaw Drop). Importantly, our findings suggest that the discrimination of surprise and fear relies on specific combinations of AUs. Thus, our approach goes beyond the analysis of single AUs and elucidates previous findings by determining the synergies of AU activations that are responsible for reliable emotion categorization.

#### Discriminating subspaces for the other four emotions

**Figure 6:**
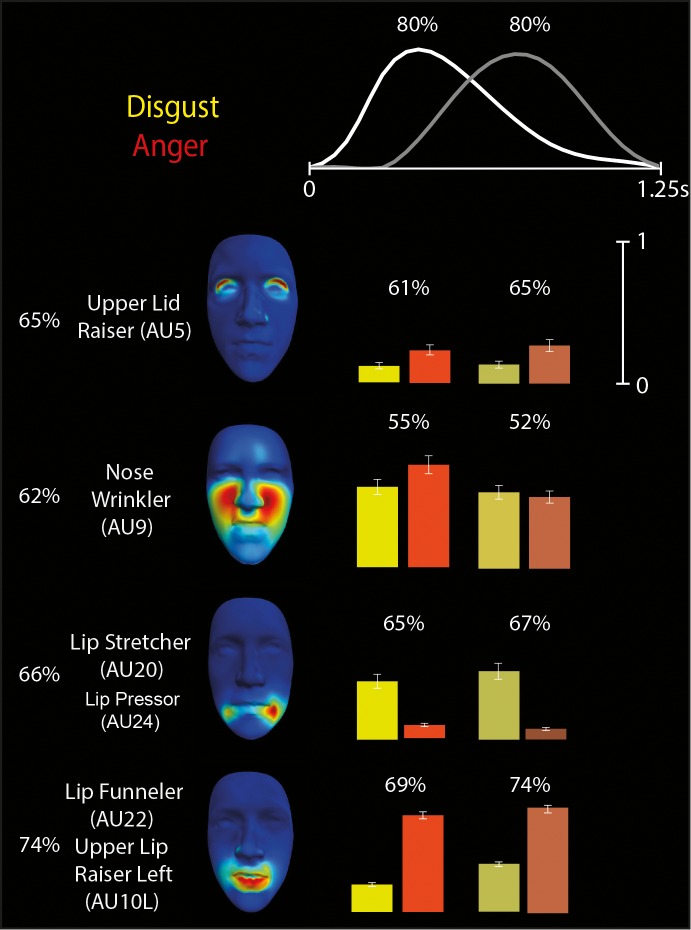
Space-by-time decomposition for disgust/anger discrimination. The space-by-time decomposition identified two temporal components (waveforms in top) and four AU components (see color-coded faces and AU labels). Color-coded bars (yellow = disgust, orange = anger, with bright colors representing activations of the first temporal component and darker colors representing activations of the second temporal component) show the activation coefficients (trial averages ± standard error of the mean) that linearly combine each temporal component with each AU component. Reported percentages correspond to the average decoding accuracy (percent correct) across observers of the spatial components (left), temporal components (top), and space-by-time linear combinations of components (on top of each color-coded pair of bars). Chance-level decoding is 50%.

**Figure 7:**
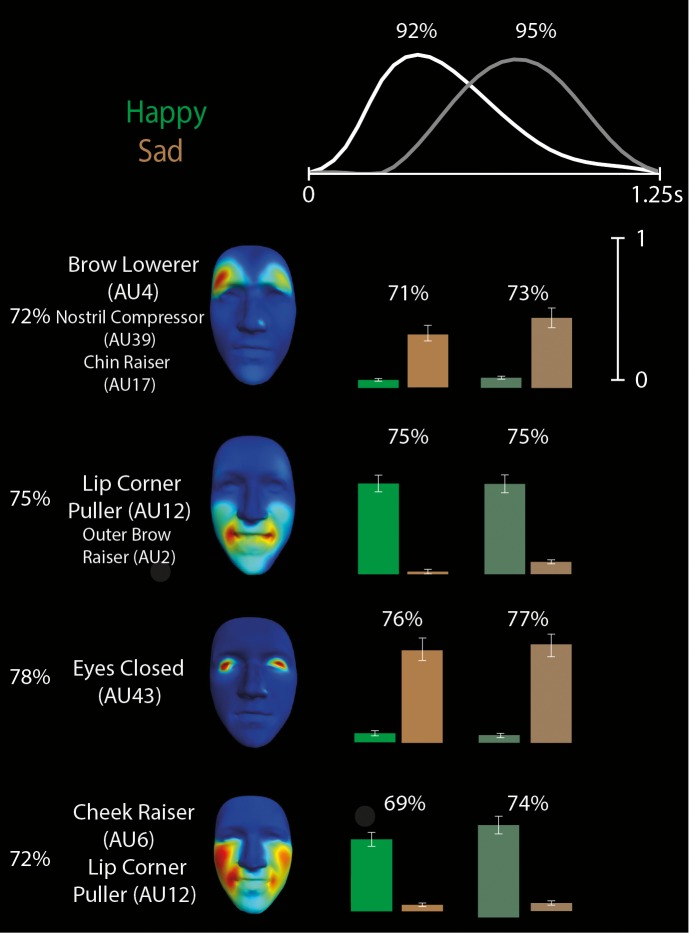
Space-by-time decomposition for happy/sad discrimination. The space-by-time decomposition identified two temporal components (waveforms in top) and four AU components (see color-coded faces and AU labels). Color-coded bars (green = happy, brown = sad, with bright colors representing activations of the first temporal component and darker colors representing activations of the second temporal component) show the activation coefficients (trial averages ± standard error of the mean) that linearly combine each temporal component with each AU component. Reported percentages correspond to the average decoding accuracy (percent correct) across subjects of the space-by-time linear combinations of components. Chance-level decoding is 50%.

We then derived space-by-time manifold representations of the diagnostic patterns of AU activation that underlie discrimination of the other four emotion categories: happy, disgust, anger, and sad. Figures 6 and 7, respectively, illustrate the space-by-time manifold representations for the disgust/anger pair, which is also typically confused (Ekman, 1992; Gagnon et al., 2010), and the happy/sad pair. As shown in Figure 6, we found that disgust and anger share the dimension of Nose Wrinkler (AU9), thereby predicting confusions between these two emotions. In contrast, the synergistic movements of Lip Stretcher–Cheek Raiser Left (AU20-6L) discriminate disgust from anger, and the synergistic movements of Lip Funneler–Upper Lip Raiser Left (AU22-10L) discriminate anger from disgust. As shown in Figure 7, happy and sad categorization shows the least confusion. Whereas happy is discriminated from sad most reliably on the basis of Lip Corner Puller–Outer Brow Raiser–Cheek Puffer (AU12-2), sad is discriminated from happy on the basis of Eyes Closed (AU43).

These components represent the AU information that the human categorizer must attend to, code, and then use to discriminate facial expressions of emotion. The main insights gained by our computational approach are, first, the dissociation between the representations of synergistic AUs into individual components, and second, a categorization decision rule that is based on the linear combination of few dimensions.

#### Relative contribution of AU and temporal components

We have shown that different AU components contribute to the categorization of the six classic emotions (see percentages to the left of AU components on Figures 4, 6, and 7). An interesting question regards the contribution of differences in the temporal-activation profiles to emotion categorization—i.e., whether the two temporal components carried similar or complementary information for emotion categorization. In Figure 3C, the confusion matrix for the six-emotion discrimination, averaged over all observers and trials, illustrates the case for using both temporal components; the confusion matrices shown in Supplementary Figure S1 illustrate the case for using only one temporal component. For the discrimination of some specific emotions, we found a clear advantage to using both temporal components. For example, using both temporal components produced a larger decoding advantage for surprise (77% ± 1% for the two components together versus 73 ± 1% and 70% ± 1%, respectively, for the early and late ones; six-emotion discrimination) and fear (54% ± 2% for the two components together versus 50% ± 2% and 43% ± 2%, respectively, for the early and late components; six-emotion discrimination). However, on average across all emotions, we found that computing the decoding accuracy of each temporal component using only the early or late component produced a similar emotion-decoding accuracy (61% ± 1% vs. 63% ± 1% for six-emotion discrimination). The decoding accuracy obtained with one component alone was less (*p* < 0.05, permutation test) than the decoding accuracy obtained with both components (66% ± 1%), but only by a relatively small amount.

Thus, our results suggest that using only either of the two would be almost enough to extract the emotion-categorization information from the data. Our hypothesis is that this redundancy is largely due to the stimulus-generation procedure. The single-trial facial animations we used here comprised largely overlapping temporal profiles that spanned most of the duration of the facial animation. As a result, both temporal components had to be activated to approximate the full temporal profiles of AUs in single trials. Therefore, the two temporal components often carried redundant information about the categorization responses. To address this issue and investigate whether temporal dynamics may indeed play a more important role in emotion categorization, we designed a validation experiment in which we manipulated stimulus dynamics to make temporal differences more pronounced.

## Experiment 2: Perceptual validation of the low-dimensional representations

To test the perceptual validity of the extracted representational manifold, we used a new set of observers to categorize the facial expressions generated by directly combining the identified AU and temporal components.

### Observers

We recruited 10 Western White observers (five women, five men; mean age = 23 years, *SD* = 2.4 years) using the same inclusion and exclusion criteria as in Experiment 1.

### Stimuli

We generated facial-expression stimuli based on the space-by-time manifold that characterizes all six emotion categories. For each emotion, we generated stimuli using the discriminating AU components determined by the LDA analysis (see Decoding analysis to identify discriminating dimensions, earlier). We further validated the temporal components by directly testing the sequencing of the discriminating AU components. Specifically, we first generated what we call the original sequence of stimulus activation, which was the sequence in which we combined each AU component with the temporal component with the maximum average activation coefficient, setting the other temporal component to 0. In the inverted sequence of stimuli, we combined each AU component with the temporal component with the lower average activation, setting the other to 0. This manipulation generated stimuli with a different dynamical structure that enabled direct and systematic investigations of how the temporal dimensions of the activation of the AUs may add independent information about emotion categorization that can affect categorization speed and accuracy. Specifically, for each emotion we produced two dynamics (original and inverted), for a total of 12 dynamics. We then applied the 12 dynamics to a total of 50 different same-race face identities (White; 25 men, 25 women; mean age = 23 years, *SD* = 4.1 years), each captured using standard procedures (for details, see Yu et al., 2012), resulting in a total of 600 facial animations.

### Procedure

Observers performed a two-alternative forced-choice task as follows. On each trial, observers first viewed an emotion label (happy, surprise, fear, disgust, anger, or sad) for 1 s. Following a 0.5-s interval, observers viewed a facial animation and indicated whether the previously presented emotion label accurately described the presented animation. We instructed observers to respond as fast and as accurately as possible by pressing one of two keyboard buttons, corresponding to yes and no. We assigned each response key to a different hand and counterbalanced key assignments across observers. The next trial started immediately following response.

For each emotion label, observers saw 50 matching facial animations with original dynamics, 50 matching facial animations with inverted dynamics, 50 nonmatching facial animations with original dynamics (10 for each other emotion), and 50 nonmatching facial animations with inverted dynamics—i.e., a total of 200 animations per emotion—for a total of 1,200 trials. We split the experiment into three sessions of 400 trials each, themselves split into eight blocks of 50 trials each. We randomized the order of trial presentation across the experiment for each observer.

### Results

#### Validation of space-by-time manifold representation

We first assessed the dependence of emotion-recognition accuracy on the presented emotion and the stimulus temporal dynamics (original or inverted). We performed a two-way analysis of variance on the response-accuracy results using emotion and dynamics (original or inverted) as factors, and found a main effect by emotion category, *F*(5, 108) = 10.91, *p* < 0.01, and dynamics, *F*(1, 108) = 12.63, *p* < 0.0001, with a statistically significant interaction, *F*(5, 108) = 3.62, *p* < 0.01.

**Figure 8:**
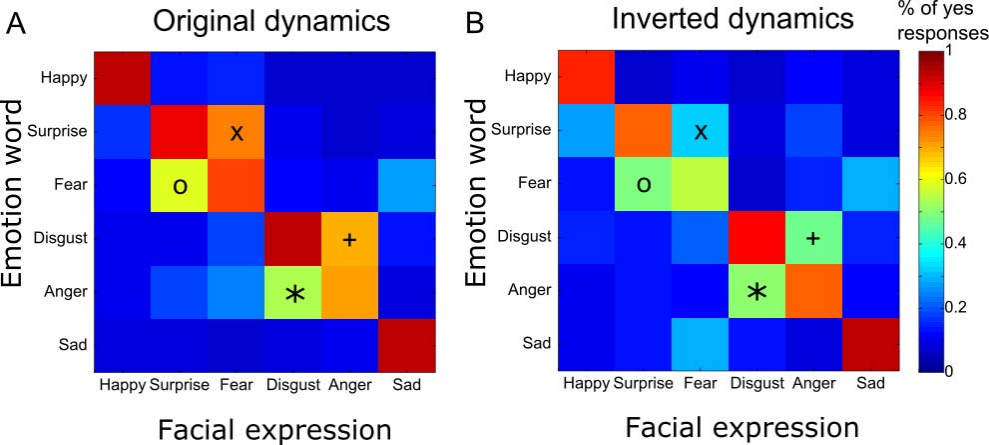
Emotion-word and facial-animation matching responses. Each color-coded matrix shows the proportion of trials where observers responded yes—i.e., they perceived that the emotion word and facial expression matched—when the facial animations had the original dynamics (A) or the inverted dynamics (B). Rows represent the emotion words presented at the beginning of the trial, and columns represent the emotions of the facial expressions presented on the same trial. Symbols X, ○, +, and * indicate the four most usual errors involving the pairs of emotions that are typically confused, namely surprise/fear and disgust/anger.

We then tested whether observers could reliably perceive the identified space-by-time manifold dimensions as the corresponding emotions. To this end, we examined observer performance on the correct matching of facial animations and emotion labels. In Figure 8, color-coded matrices show the results, where rows correspond to the emotion labels and columns correspond to the facial animations. Color-coded squares in each matrix show the proportion of trials where observers responded yes for original (Figure 8A) and inverted (Figure 8B) dynamics (see color bar to the right). As shown by the red squares across the diagonal in both matrices, observers correctly matched the facial animations and emotion labels on the vast majority of occasions for most emotion categories. For animations presented with the original dynamics, observers typically performed a correct word–stimulus match (87% average hit rate computed across all six emotions). When the animations and emotion labels did not match, observers rarely responded incorrectly—i.e., yes (20% average false-alarm rate, see off-diagonal values). For the original dynamics, observers performed significantly more accurately (Tukey's multiple comparison test, all *p*s < 0.05) for happy (94% ± 2%), surprise (89% ± 4%), disgust (93% ± 1%), and sad (93% ± 1%) than anger (72% ± 5%).

We then investigated whether the dynamics affected the emotion-matching accuracy. We found that for four emotions (happy, surprise, fear, and disgust) the original temporal sequence elicited significantly more hits (paired *t* tests, all *p*s < 0.05) than the inverted sequence (higher diagonal values in Figure 8A than in Figure 8B)—happy: 94% ± 2% versus 84% ± 4%, *t*(18) = 2.36; surprise: 89% ± 4% versus 77% ± 4%, *t*(18) = 2.23; fear: 80% ± 3% versus 57% ± 8%, *t*(18) = 2.74; and disgust: 93% ± 1% versus 88% ± 2%, *t*(18) = 2.15. This suggests that temporal sequencing of AU components is important for the recognition of these emotions. Anger is the only emotion that showed slightly but not significantly higher hit rates for the inverted temporal dynamics than the original dynamics: 78% ± 4% versus 72% ± 5% (paired *t* test, *p* = 0.37), *t*(18) = −0.92.

We then considered pairs of emotions that were typically confused, namely surprise/fear and disgust/anger, and observed that these confusions were asymmetric, in agreement with previous findings (Du & Martinez, 2011). Specifically, surprise had more false alarms than fear (X and O in Figure 8A) and disgust more than anger (+ and * in Figure 8A). In other words, fearful facial expressions were incorrectly matched with surprise labels significantly more often than vice versa—75% ± 5% versus 59% ± 9% (paired *t* test, *p* < 0.05), *t*(9) = 2.18—and angry facial expressions were more often (paired *t* test, *p* < 0.05) matched with disgust labels than vice versa: 70% ± 8% versus 54% ± 8%, *t*(9) = 2.31. Interestingly, when the temporal dynamics of the facial animations were inverted, the number of these confusions decreased significantly for surprise false alarms—39% ± 8% versus 75% ± 5% (paired *t* test, *p* < 0.0001), *t*(9) = 5.9, (X in Figure 8A, B)—and disgust false alarms: 47% ± 8% versus 70% ± 8% (paired *t* test, *p* < 0.01), *t*(9) = 3.1 (+ in Figure 8A, B). It did not decrease significantly for fear—49% ± 7% versus 59% ± 9% (paired *t* test, *p* = 0.43), *t*(9) = 0.83 (O in Figure 8A, B)—and anger: 50% ± 9% versus 54% ± 8% (paired *t* test, *p* = 0.69), *t*(9) = 0.41 (* in Figure 8A, B).

To explain these results, we identified the temporal sequences of AU components that are responsible for the observed confusions and those that resolve the confusions. In particular, the original facial animations contained an early activation of Upper Lid Raiser (AU5) for surprise and fear and of Nose Wrinkler (AU9) for disgust and anger. When the temporal sequence was inverted, these AUs appeared later. Upper Lid Raiser (AU5) was preceded by Mouth Stretch–Outer Brow Raiser (AU27-2) for surprise and Lip Stretcher–Cheek Raiser Left (AU20-6L) for fear, and Nose Wrinkler (AU9) was preceded by Lip Stretcher–Cheek Raiser Left (AU20-6L) for disgust and Lip Funneler–Upper Lip Raiser Left (AU22-10L) for anger. Hence, the decrease in confusions when the inverted dynamics were used confirms our previous finding that confusions are due to Upper Lid Raiser (AU5) and Nose Wrinkler (AU9), suggesting that the AU components that typically arise later in time contain diagnostic information for distinguishing fear from surprise and anger from disgust. This finding is consistent with a hierarchical organization of facial-expression signals over time (Jack et al., 2014).

These findings demonstrate the usefulness of the space-by-time decomposition in describing facial expressions of emotions by determining synergies between AUs in space and time that code each emotion.

#### Temporal dynamics affects the speed of emotion categorization

We then aimed to quantify the dependence of response times on the dynamics of the facial animations presented and the observer's responses. We performed a three-way analysis of variance on response times using as main factors emotion, dynamics (original or inverted), and response (yes or no). We found main effects of emotion, *F*(5, 11976) = 9.78, *p* < 0.0001, dynamics, *F*(1, 11976) = 3.91, *p* < 0.05, and response, *F*(1, 11976) = 14.47, *p* < 0.0001, with a significant interaction between emotion and dynamics, *F*(5, 11976) = 5.54, *p* < 0.0001.

**Figure 9:**
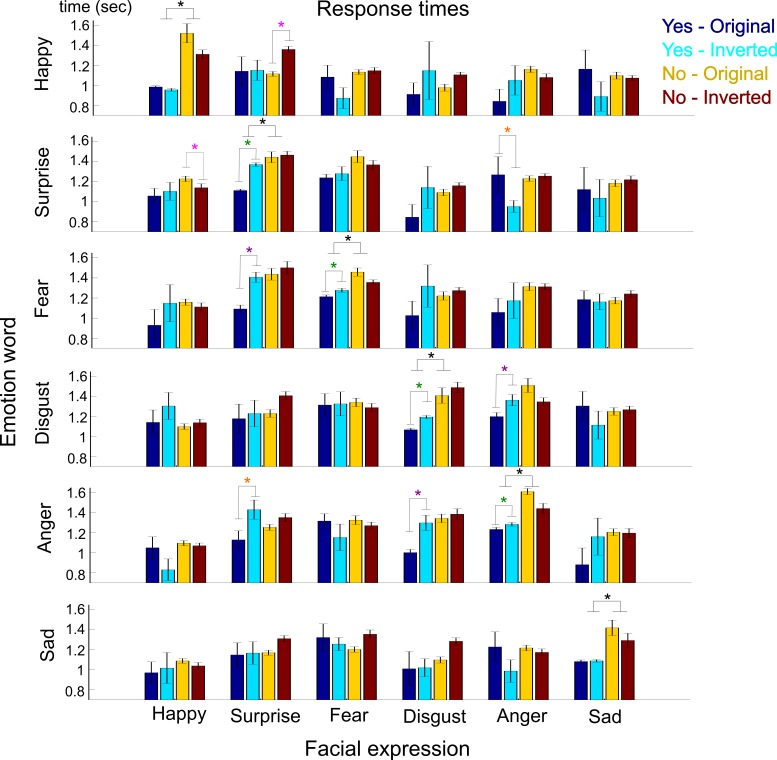
Response times to matching task of emotion words and facial expressions. Color-coded bars show the response times (average ± standard error of the mean) for the four different combinations of stimulus dynamics and responses (see legend in top right). Rows represent the emotion words presented at the beginning of the trial, and columns represent the emotions of the facial expressions presented on the same trial. Black stars indicate significant differences in response times between hits and misses (*p* < 0.01). Color-coded stars indicate significant differences in response times as a result of the inversion of the stimulus temporal dynamics (*p* < 0.05).

First, we observed a strong dependence of response times on the correctness of the observers' responses. In particular, for all emotion categories, hits had significantly faster (Tukey's multiple comparison test, *p* < 0.01) reaction times than misses (see Table 1 and black stars in Figure 9).

**Table 1:**
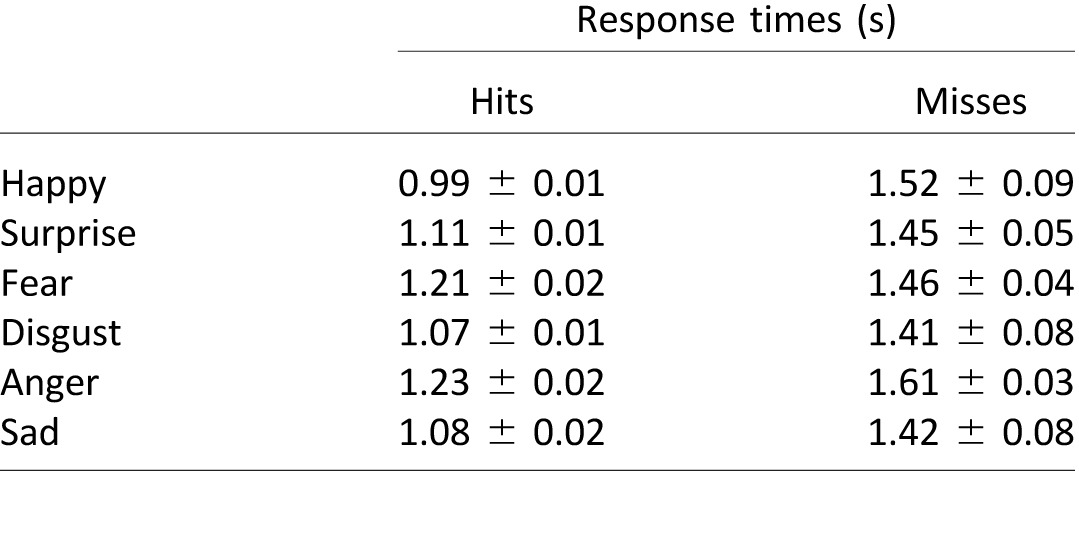
Average response times (± standard error of the mean) for hits and misses for each emotion category.

Second, in order to investigate how the temporal sequencing of the presented stimulus affects the recognition of facial expressions, we assessed the dependence of response times on the stimulus dynamics. We found that inverting the dynamics resulted in significantly slower (paired *t* tests, all *p*s < 0.05) response times for hit trials for surprise: 1.11 ± 0.01 s versus 1.37 ± 0.02 s on average, *t*(816) = −11.97; fear: 1.21 ± 0.02 s versus 1.28 ± 0.02 s on average, *t*(676) = −2.46; disgust: 1.07 ± 0.01 s versus 1.20 ± 0.02 s on average, t(891) = −5.99; and anger: 1.23 ± 0.02 s versus 1.29 ± 0.02 s on average, *t*(740) = −2.10 (green stars in Figure 9). It had no effect on happy or sad hit trials, suggesting that correct matching of surprise, fear, disgust, and anger depends on the temporal sequencing of AU activations, while the stimulus dynamics do not affect correct matching of happy or sad. This result confirms previous findings showing that happy and sad are reliably categorized early on, whereas the other four emotions are discriminated by means of AU signals transmitted later in time (Jack et al., 2014).

We summarize the response-time results in Figure 9, where each row represents one of the six emotion labels and each column represents one of the six facial expressions presented on each trial. Color-coded bars show the average response times (± standard error of the mean) depending on the observers' responses (yes or no) and stimulus dynamics (original or inverted).

#### Differences in response times reveal diagnostic sequences of AU components

**Figure 10:**
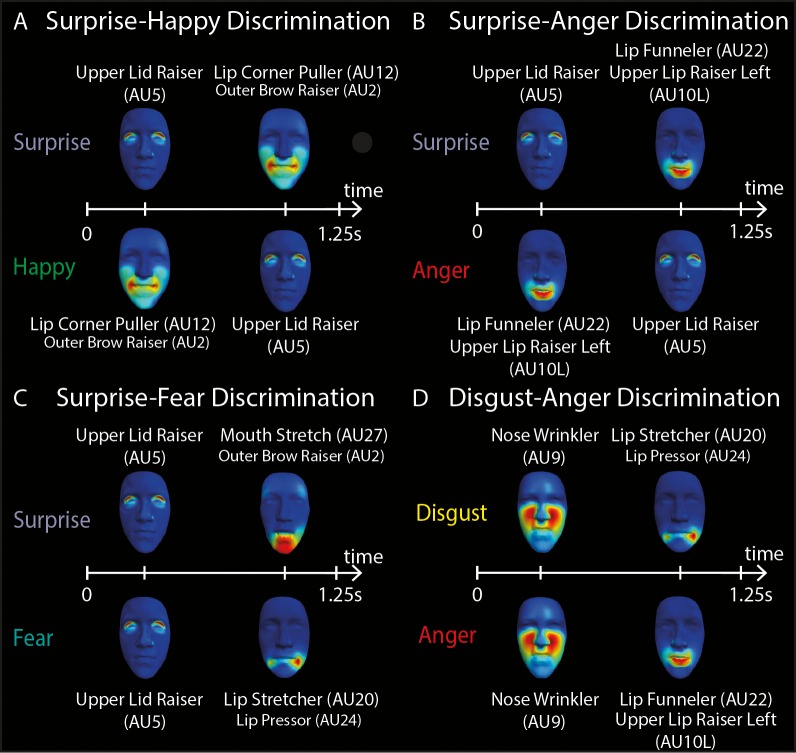
Diagnostic temporal sequences of AU components. A schematic illustration of the temporal dynamics of AU components that discriminate (A) surprise and happy, (B) surprise and anger, (C) surprise and fear, and (D) disgust and anger. In each panel, the color-coded faces represent AU components (see AU labels below each face) that are activated sequentially in time (from 0 to 1.25 s, see white horizontal line) to discriminate between two emotions (see emotion words to the left).

Finally, we examined whether the observed differences in response time could be due to the sequencing of AU components. We hypothesized that response times would depend highly on the early or late activation of diagnostic or confusing AUs. Hence, we assessed the dependence of response times on the AU sequences in order to uncover the AUs that provide conflicting or diagnostic information for emotion categorization. Indeed, we identified significant response-time differences that informed the role of specific AU components and their sequencing on emotion recognition. Figure 10 provides a summary of our findings by illustrating the temporal sequences of AUs that discriminate between emotion pairs.

For instance, when observers viewed a happy label followed by surprised facial expressions, correct rejections depended on stimulus dynamics. Specifically, inverting the dynamics resulted in slower response times for correct rejections compared to the original dynamics—1.36 ± 0.03 s versus 1.12 ± 0.02 s on average (paired *t* test, *p* < 0.05), *t*(154) = 2.16 (pink star in Figure 10)—suggesting a reliance on the late activation of Upper Lid Raiser (AU5). Conversely, when the surprise label was shown followed by a happy facial expression, correct rejection relied on Lip Corner Puller–Outer Brow Raiser (AU12-2), because its early presentation (in the inverted dynamics) shortened response time: 1.14 ± 0.03 s versus 1.23 ± 0.04 s on average (paired *t* test, *p* < 0.0001), *t*(177) = −6.24 (pink star in Figure 10). Thus, Upper Lid Raiser (AU5) and Lip Corner Puller–Outer Brow Raiser (AU12-2) provide diagnostic information to discriminate happy and surprise (Figure 10A).

Similarly, we found a set of AU components that cause confusions between surprise and anger and determined a temporal sequence that resolves them. Specifically, we observed that when the surprise label appeared before an anger facial expression, false alarms depended on dynamics. Lip Funneler–Upper Lip Raiser Left (AU22-10L) drove these confusions, as earlier activation of this component shortened response time: 0.95 ± 0.06 s versus 1.27 ± 0.18 s on average (paired *t* test, *p* < 0.05), *t*(22) = 2.20 (orange star in Figure 10). Conversely, when the anger label was shown followed by a surprise facial expression, false alarms occurred due to Upper Lid Raiser (AU5): 1.13 ± 0.09 s for original dynamics versus 1.43 ± 0.10 s for inverted dynamics on average (paired *t* test, *p* < 0.05), *t*(368) = −2.16 (orange star in Figure 10). Thus, activations of Lip Funneler–Upper Lip Raiser Left (AU22-10L) and Upper Lid Raiser (AU5) caused confusions between surprise and anger. An early dynamics of Upper Lid Raiser (AU5) for surprise and a later one for anger resolves these confusions. Likewise, an early Lip Funneler–Upper Lip Raiser Left (AU22-10L) for anger and a later one for surprise resolves confusions (Figure 10B).

Analysis of the dependence of response times on the stimulus dynamics also explains the typical surprise/fear and disgust/anger confusions and identifies diagnostic sequences of AU components. When the fear label appeared before a surprise facial expression, false alarms depended on Upper Lid Raiser (AU5). When the disgust label appeared before an anger facial expression, false alarms depended on Nose Wrinkler (AU9). (See purple stars in Figure 9.) In these cases, later AU activations (for inverted dynamics) elicited slower response times(paired *t* tests, all *p*s < 0.01)—fear false alarms: 1.09 ± 0.04 s versus 1.41 ± 0.04 s on average, *t*(105) = −5.10; disgust false alarms: 1.20 ± 0.04 s versus 1.36 ± 0.06 s on average, *t*(113) = −2.64; and anger false alarms: 1.00 ± 0.03 s versus 1.30 ± 0.08 s on average, *t*(100) = −3.69. Taken together with the categorization results presented earlier, these results corroborate that surprise/fear and disgust/anger confusions are caused by the sharing of AU components (Figure 10C, D).

## Discussion

We developed and applied a novel computational framework to identify the low-dimensional information structures (i.e., manifolds) that the brain uses to categorize dynamic facial expressions according to the six classic emotions. Within this framework, we characterized dynamic emotion categorization as a factorization problem in space and time, with an initial dimensionality-reduction stage that codes the main AU and temporal synergies of facial expressions (the spatial and temporal components). In this low-dimensional manifold, we found linear boundaries that discriminate the six classic emotions and validated the low-dimensional AU components as diagnostic of the six emotions. Finally, we tested the role of facial-movement dynamics on categorization accuracy and speed, showing that typical confusions of fear and surprise and of disgust and anger are caused by shared AU activations, which can be resolved by altering the sequencing of the AU components.

### Higher order reverse correlation

Our approach extends typical reverse-correlation and classification-image approaches (Murray, 2011). Whereas typical reverse-correlation analyses are first order, applying independently to each dimension of the stimulus space, our approach captures higher order correlations in the stimulus space and thus reduces it to few dimensions of categorization. Furthermore, our approach computes which of these single dimensions or linear combinations of dimensions reliably describe human categorizations. Our application suggested that few synergistic AU and temporal components form the dimensions that suffice to categorize each of the six classic emotions—when the initial stimulus space is high dimensional with 252 degrees of freedom.

A method that identifies a generative space of facial expressions where emotions can be discriminated has been proposed recently (Martinez & Du, 2012). This method differs from our approach in important aspects. First, it implements a discriminant analysis to identify the shape and configural facial features that discriminate each emotion from all others. Instead, we implement a dimensionality-reduction method to identify the informative manifold dimensions in the space defined by dynamic AUs and then determine the manifold regions where emotions are reliably discriminated. Second, the Martinez & Du model defines emotion-specific components from which new emotion categories can be generated as linear combinations of the six emotions. In contrast, our approach identifies components that are shared across the six emotions, and each emotion is defined distinctly as a linear combination of the components. Future work is required to test whether the components we found here can be used as a generative space for new emotion categories as well. Also, further studies could aim to map the two manifolds, particularly the emotion-discriminating subspaces extracted by the two approaches.

### Categorization manifolds, information structure, and optimal signal design

Our method also serves to explain the information structure of categorization, by specifying the information of correct categorizations and their confusions (Houlsby et al., 2013; Ullman, Vidal-Naquet, & Sali, 2002). For example, we found that the Upper Lid Raiser (AU5) caused the typical confusions between fear and surprise (Jack et al., 2014). We also found that this confusion is reduced when Mouth Stretch and Outer Brow Raiser (AU27-2) categorize surprise and Upper Lid Raiser (AU5) in conjunction with slightly delayed Lip Stretcher (AU20) and Cheek Raiser Left (AU6L) categorizes fear. Relatedly, whereas Nose Wrinkler (AU9) causes confusions between disgust and anger, we found that combining this AU with Lip Stretcher (AU20) and Cheek Raiser Left (AU6L) leads to discrimination of disgust (Jack et al., 2014), whereas combining it with Lip Funneler (AU22) and Upper Lip Raiser Left (AU10L) leads to discrimination of anger. Consequently, this information enables the design of optimal social signals by minimizing their confusions.

### Holistic coding dimensions and categorization

As highlighted earlier, our approach predicts that the incoming stimulus is initially projected on unitary dimensions that code AU synergies and temporal synergies before being linearly classified. Psychologically speaking, this places the burden of the categorization problem onto the coding stage, because each higher order coding dimension must be learned before the projection of the stimulus can be effectively used for linear classification. This raises the question of how the dimensions themselves are learned over the course of psychological development (Schyns, Goldstone, & Thibaut, 1998). It also raises an interesting point for studies of attention. The existence of unitary dimensions suggests that the higher order information they comprise is coded holistically—and that in fact it is the synergistic movements that elicit their unitary representation. Thus, we would predict that each dimension constitutes an “atom” of coding in the cognitive architecture. Notably, Jack et al. (2014) showed that Nose Wrinkler (AU9) and Upper Lid Raiser (AU5) occur early in the AU time series, each with early peak activations. They constitute unitary dimensions, suggesting that these two AUs are isolated from the subsequent facial movements comprising the expressions. The consequences of this observation for attention and stimulus coding will be the object of further studies.

### Spatial frequencies and facial movements

We have previously shown that the human face transmits facial expressions of emotion over a range of viewing distances (Smith & Schyns, 2009). On the basis of the spatial-frequency spectrum of the classic facial expressions studied here, we found a gradient of categorization generalization, whereby performance decreased with viewing distances in the following order: sad, anger, fear, disgust, surprise, and happy. Thus, happy and surprise were the most distal expressions, on the basis of the lower spatial-frequency representation of their diagnostic information (the opened mouth), whereas sad and anger were the most proximal expressions, on the basis of the comparatively higher spatial-frequency representation of their diagnostic information. Here, though we did not test categorization over viewing distances, it must be noted that the dynamics of facial movements can add diagnostic information for their categorization. How dynamic information between the onset of movement and its peak amplitude facilitates or impairs expression categorization over a range of viewing distances will be the object of further studies.

### Generative spaces for other social signals and stimulus categories

Our approach is not restricted to the categorization of emotions. It can be straightforwardly extended to a broad range of categorization problems, especially when involving a stimulus-generation space that is reasonably well understood. For example, using dynamic bodies, our approach could extend to characterize the categorization information of the expression revealed by different types of gait (e.g., walking vs. running). Using static 3-D faces, we could also extract the underlying structure for categorizing ethnicity, gender, or identity. In contrast, our approach would be less applicable to complex stimuli that are not readily decomposable into their generative components (as temporal AUs are generative in our example). Such complex stimuli include scenes, where the generative components are notoriously difficult to isolate—e.g., are they the objects of the scenes, their parts, or some scene properties themselves, and if so, which ones? Understanding the generative elements of faces, objects, and scenes precedes the application of our generative framework, and it is this understanding that must become a focus of future research.

## Supplementary Material



Supplement 1
