# Supplementary figures and images for: Space-by-time manifold representation of dynamic facial expressions for emotion categorization
Source: J Vis. 2016 Jun 15;16(8):14. doi: 10.1167/16.8.14 (PMC4927208; doi:10.1167/16.8.14)

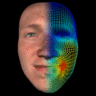

Supplement: Supplementary file 1 [file i1534-7362-16-8-14-icon.gif]
