# Supplementary material for: Space-by-time manifold representation of dynamic facial expressions for emotion categorization
Source: J Vis. 2016 Jun 15;16(8):14. doi: 10.1167/16.8.14 (PMC4927208; doi:10.1167/16.8.14)
Supplement: Supplement 1 [file i1534-7362-16-8-14-s01.pdf]

Supplementary Material

Space-by-time manifold representation of dynamic facial expressions for emotion categorization

Ioannis Delis[1,2]*, Chaona Chen[3], Rachael E. Jack[1,3], Oliver G. B. Garrod[1], Stefano Panzeri[4], Philippe G. Schyns[1,3].

[1]Institute of Neuroscience and Psychology, School of Psychology, University of Glasgow, 58 Hillhead Street, Glasgow G12 8QB, United Kingdom

[2]Department of Biomedical Engineering, Columbia University, New York, NY 10027, USA

[3]School of Psychology, University of Glasgow, Glasgow G12 8QB, United Kingdom

[4]Laboratory of Neural Computation, Center for Neuroscience and Cognitive Systems @UniTn, Istituto Italiano di Tecnologia, Via Bettini 31, 38068 Rovereto (TN), Italy

***Correspondence to: ioannis.delis@columbia.edu**

Keywords: emotion categorization, space-by-time manifold, NMF, mental representations, dynamic facial expressions

Facial Action Units

The 42 Action Units (AUs) - representing specific facial movements performed by specific facial muscle[s] as described by the Facial Action Coding System [FACS] (Ekman, Friesen, & Hagar, 1978)- that we used to generate the facial expression stimuli used in our experiments are shown in Supplementary Table 1. All AUs are unilateral except for Cheek Raiser (AU6), Lid Tightener (AU7), Upper Lip Raiser (AU10), Lip Corner Puller (AU12), Dimpler (AU14), and Lip Stretcher (AU20) which are bilateral.

Inner Brow Raiser	AU1
Inner-Outer Brow Raiser	AU1-2
Outer Brow Raiser	AU2
Outer Brow Raiser Left	AU2L
Brow Lowerer	AU4
Upper Lid Raiser	AU5
Cheek Raiser	AU6
Cheek Raiser Left	AU6L

Cheek Raiser Right	AU6R
Lid Tightener Left	AU7L
Lid Tightener Right	AU7R
Nose Wrinkler	AU9
Upper Lip Raiser	AU10Open
Upper Lip Raiser Left	AU10LOpen
Upper Lip Raiser Right	AU10ROpen
Nasolabial Deepener Left	AU11L
Nasolabial Deepener Right	AU11R
Lip Corner Puller	AU12
Lips Part-Lip Corner Puller	AU12-25
Lip Corner Puller Left	AU12L
Lip Corner Puller Right	AU12R
Sharp Lip Puller	AU13
Dimpler	AU14
Dimpler Left	AU14L
Dimpler Right	AU14R
Lip Corner Depressor	AU15
Lower Lip Depressor	AU16Open
Chin Raiser	AU17
Lip Stretcher	AU20
Lip Stretcher Left	AU20L
Lip Stretcher Right	AU20R
Lip Funneler	AU22
Lip Tightener	AU23
Lip Pressor	AU24
Lips Part	AU25
Jaw Drop	AU26
Mouth Stretch	AU27i
Nostril Dilator	AU38
Nostril Compressor	AU39
Eyes Closed	AU43
Lid Tightener	AU7
Cheek Raiser-Lip Corner Puller	AU6-12

Supplementary Table1:The 42 Action Units.

Stimulus parameter sampling

For each observer, we generated 2400 facial animations by selecting a subset of AUs and random values for the six temporal parameters. Although, the resulting stimuli cannot cover all possible combinations of AUs and temporal parameters, we selected the parameters in order to cover as much of the stimulus space as possible space using the following procedure. For the 2400 trials of each observer, we ensured that all AUs were active in approximately equal subsets of trials (each AU was active in 159 of the trials on average) and that all pairs of AUs were presented in 10 trials on average. On each trial, all temporal parameters were independently sampled uniformly from 0 to 1. Hence, given the above statistics, if we split the values of each parameter into e.g. 3 bins (0-0.33, 0.33-0.67, 0.67-1), each parameter bin appeared in around 53 (out of 159) trials for each AU. Onset latency, offset latency, acceleration and deceleration were scaled relative to peak latency in order to produce meaningful temporal waveforms representing plausible facial movements (see Yu, Garrod, & Schyns, 2012 for details).

Screening Questionnaire

Each potential observer completed the following questionnaire. We selected only those answering ‘no’ to all questions for participation in the experiment.

1. Have you ever lived in non-Western* country before (e.g. on a gap year, summer work, move due parental employment)?
2. How many weeks have you spent in a non-Western country (e.g. on vacation)?
3. Have you ever dated or had a very close friendship with a non-Western person?
4. Have you ever been involved with any non-Western culture societies/groups?

*by Western groups/countries, we are referring to Europe (East and West), USA, Canada, United Kingdom, Australia and New Zealand.

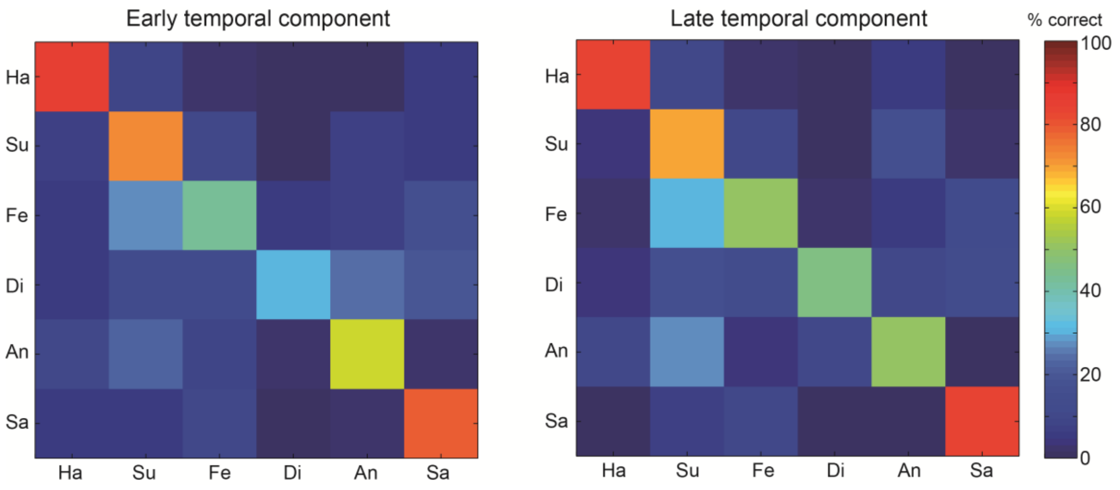

Supplementary Figure1: The confusion matrices (averaged across subjects) for the discrimination of the six emotions using only the early temporal component (left) and only the late temporal component (right).

References

Yu, H., Garrod, O. G. B., & Schyns, P. G. (2012). Perception-driven facial expression synthesis. *Computers & Graphics-Uk, 36*(3), 152-162.

Ekman, P., Friesen, W., & Hagar, J. C. (Eds.). (1978). *Facial Action Coding System Investigators Guide*. Salt Lake City: Research Nexus.
